# Fast skeletal muscle troponin activator CK‐2066260 mitigates skeletal muscle weakness independently of the underlying cause

**DOI:** 10.1002/jcsm.12624

**Published:** 2020-09-21

**Authors:** Arthur J. Cheng, Jennifer Ström, Darren T. Hwee, Fady I. Malik, Håkan Westerblad

**Affiliations:** ^1^ Department of Physiology and Pharmacology Karolinska Institutet Stockholm Sweden; ^2^ School of Kinesiology and Health Science, Faculty of Health York University Toronto Canada; ^3^ Research and Early Development Cytokinetics, Inc. South San Francisco CA USA

**Keywords:** Muscle weakness, Troponin activator, Free cytosolic [Ca^2+^], Prolonged low‐frequency force depression, Muscle fatigue

## Abstract

**Background:**

Muscle weakness is a common symptom in numerous diseases and a regularly occurring problem associated with ageing. Prolonged low‐frequency force depression (PLFFD) is a form of exercise‐induced skeletal muscle weakness observed after exercise. Three different intramuscular mechanisms underlying PLFFD have been identified: decreased sarcoplasmic reticulum Ca^2+^ release, decreased myofibrillar Ca^2+^ sensitivity, and myofibrillar dysfunction. We here used these three forms of PLFFD as models to study the effectiveness of a fast skeletal muscle troponin activator, CK‐2066260, to mitigate muscle weakness.

**Methods:**

Experiments were performed on intact single muscle fibres or fibre bundles from mouse flexor digitorum brevis, which were stimulated with electrical current pulses, while force and the free cytosolic [Ca^2+^] ([Ca^2+^]_i_) were measured. PLFFD was induced by three different stimulation protocols: (i) repeated isometric contractions at low intensity (350 ms tetani given every 5 s for 100 contractions); (ii) repeated isometric contractions at high intensity (250 ms tetani given every 0.5 s for 300 contractions); and (iii) repeated eccentric contractions (350 ms tetani with 20% length increase given every 20 s for 10 contractions). The extent and cause of PLFFD were assessed by comparing the force–[Ca^2+^]_i_ relationship at low (30 Hz) and high (120 Hz) stimulation frequencies before (control) and 30 min after induction of PLFFD, and after an additional 5 min of rest in the presence of CK‐2066260 (10 μM).

**Results:**

Prolonged low‐frequency force depression following low‐intensity and high‐intensity fatiguing contractions was predominantly due to decreased sarcoplasmic reticulum Ca^2+^ release and decreased myofibrillar Ca^2+^ sensitivity, respectively. CK‐2066260 exposure resulted in marked increases in 30 Hz force from 52 ± 16% to 151 ± 13% and from 6 ± 4% to 98 ± 40% of controls with low‐intensity and high‐intensity contractions, respectively. Following repeated eccentric contractions, PLFFD was mainly due to myofibrillar dysfunction, and it was not fully reversed by CK‐2066260 with 30 Hz force increasing from 48 ± 8% to 76 ± 6% of the control.

**Conclusions:**

The fast skeletal muscle troponin activator CK‐2066260 effectively mitigates muscle weakness, especially when it is caused by impaired activation of the myofibrillar contractile machinery due to either decreased sarcoplasmic reticulum Ca^2+^ release or reduced myofibrillar Ca^2+^ sensitivity.

## Introduction

Muscle weakness is a common symptom in numerous diseases, and it is also a regularly occurring problem associated with ageing.^1^ Reduced muscle strength can have a large negative impact on everyday activities and quality of life, and population studies reveal an association between reduced muscle strength and mortality.^2^, ^3^ Muscle weakness is generally due to a combination of atrophy (i.e. decreased muscle cross‐sectional area) and impaired muscle quality (i.e. a reduced ability of muscle fibres to generate force).^4^ Intracellularly, impaired muscle quality can be due to decreased myofibrillar force‐generating capacity and/or impaired activation of the myofibrillar contractile machinery, which in turn can be caused by decreased sarcoplasmic reticulum (SR) Ca^2+^ release and/or reduced myofibrillar Ca^2+^ sensitivity.^5^ Prolonged muscle weakness related to reductions in myofibrillar force‐generating capacity and Ca^2+^ sensitivity due to a preferential loss of myosin has been observed in mechanically ventilated intensive care unit patients and patients with cancer cachexia.^6^, ^7^, ^8^ Similarly, impaired force‐generating capacity combined with decreased myofibrillar Ca^2+^ sensitivity has been observed in animal models of rheumatoid arthritis, but in this case, actin, rather than myosin, appears preferentially affected.^9^, ^10^, ^11^ Moreover, muscle weakness has been linked to decreased SR Ca^2+^ release in ageing^12^ as well as in several pathological conditions, including heart failure,^13^ muscular dystrophy,^14^ mitochondrial myopathy,^15^ and bone cancer.^16^

Prolonged low‐frequency force depression (PLFFD) is typically described as a long‐lasting muscle weakness that persists in the recovery period after endurance‐type exercise.^17^, ^18^, ^19^ This muscle weakness is most evident during submaximal contractions in which a given frequency of motor unit activity gives a lower than expected contractile force. Because PLFFD is readily observed with direct electrical muscle stimulation in humans after fatiguing exercise and in isolated fatigued mammalian skeletal muscle, intramuscular mechanisms have been implicated as major contributors to PLFFD.^19^, ^20^, ^21^, ^22^, ^23^, ^24^, ^25^ A phenomenon similar to PLFFD has been found after resistance‐type exercise involving high‐force eccentric contractions.^26^, ^27^ PLFFD is generally attributed to decreased SR Ca^2+^ release and/or reduced myofibrillar Ca^2+^ sensitivity after isometric contractions, whereas reduced myofibrillar force‐generating capacity may contribute to PLFFD after eccentric contractions.^18^, ^19^ Thus, PLFFD involves the same cellular mechanisms as those causing muscle weakness in several disease conditions (see earlier).

Fast skeletal muscle troponin activators (FSTAs) are small molecules that decrease the off‐rate of Ca^2+^ from troponin C, resulting in amplified force for a given submaximal myoplasmic free [Ca^2+^] ([Ca^2+^]_i_).^28^, ^29^, ^30^, ^31^ Motor units generally fire at submaximal frequencies during voluntary contractions,^32^, ^33^ and FSTAs are most effective during submaximal contractions where even minor increases in the [Ca^2+^]‐dependent activation of the contractile machinery result in large force increases due to the steep force–[Ca^2+^]_i_ relationship.^29^, ^31^ Indeed, the FSTA CK‐2066260 effectively increased force when muscle fibres had entered the steep part of the force–[Ca^2+^]_i_ relationship during fatigue induced by repeated tetanic contractions.^31^ Thus, FSTAs can prove effective in amplifying submaximal force, especially under conditions with reduced submaximal force, such as the muscle weakness accompanying several pathological conditions. The purpose of this study is to use PLFFD induced by different exercise tasks, and hence caused by different mechanisms, as a tool to assess the effectiveness of FSTAs to combat muscle weakness in various pathological conditions. Our results show that the FSTA CK‐2066260 effectively mitigates muscle weakness due to decreased myofibrillar activation due to either decreased SR Ca^2+^ release or reduced myofibrillar Ca^2+^ sensitivity.

## Methods

### Mice

All animal experiments were performed at Karolinska Institutet in the Department of Physiology and Pharmacology, and all animal experimental procedures were approved by the Stockholm North local ethical committee (N19/15) and complied with the Swedish Welfare Ordinance, and applicable regulations and recommendations from Swedish authorities. Eight‐week‐old female C57BL6jRj mice (Janvier Labs, Le Genest‐Saint‐Isle, France) were acclimated for at least 7 days before being used for experiments. Mice were housed in cages with a 12/12 h light/dark cycle and were provided with food and water *ad libitum*. A total of 13 mice were used for experiments.

Mice were killed by rapid cervical dislocation, and whole flexor digitorum brevis muscles (FDBs) were removed. Intact single muscle fibres were mechanically isolated with tendons attached, as described elsewhere.^34^ This intact single fibre preparation is appropriate for prolonged experiments as there is no, or very little, rundown in force production during procedures lasting several hours.^31^, ^35^, ^36^ For all experiments, fibres were superfused at room temperature (~26°C) using a constant rate superfusion system without recirculation, including the periods when fibres were exposed to CK‐2066260. Thus, fibres were continuously superfused with fresh oxygenated Tyrode's solution (95% O_2_ and 5% CO_2_) of the following composition (in mM): 121 NaCl, 5.0 KCl, 1.8 CaCl_2_, 0.5 MgCl_2_, 0.4 NaH_2_PO_4_, 24.0 NaHCO_3_, 0.1 EDTA, and 5.5 glucose. For the isometric contractions, single fibres were mounted on hooks between an Akers 801 force transducer (Kronex Technologies, Oakland, CA, USA) and an adjustable holder that allowed muscle length to be adjusted to the optimum length of force production. For the lengthening contractions, single fibres, or small bundles of 5–10 fibres, were mounted on hooks between an Aurora Scientific force transducer (Model 1500A, Aurora Scientific, Aurora, Ontario, Canada) and a motor lever that controlled the lengthening contractions (Model 315C, Aurora Scientific). The smallest and largest fibre diameters were measured at optimal length using a camera mounted to the inverted microscope, and these diameters were used to calculate the cross‐sectional area assuming that the fibre had an elliptical cross section. [Ca^2+^]_i_ measurements were performed by injecting fibres with indo‐1 pentapotassium salt or loading them with the membrane permeable AM dye (Thermo Fisher Scientific, Gothenburg, Sweden). Indo‐1 is a ratiometric high‐affinity calcium indicator excited at 346 nm wavelength using a Xenon light source and monochromator (Horiba Scientific, Photon Technology International, London, Canada) with light emitted at 405 and 495 nm recorded by two photomultipliers (Horiba Scientific, Photon Technology International) and calculated as a ratio. Data were acquired at a 100 Hz sampling rate. Indo‐1 fluorescence ratios were converted into [Ca^2+^]_i_ using an *in vivo* calibration as described previously.^34^ During each contraction, [Ca^2+^]_i_ was measured as the mean over the entire 350 ms stimulation period.

### Stimulation protocols

Supramaximal current pulses with a duration of 0.5 ms were used to stimulate muscle fibres to contract. Fibres were exposed to one of the three stimulation protocols: (i) low‐intensity isometric contractions, (ii) high‐intensity isometric contractions, and (iii) eccentric contractions (see Supporting Information, *Figure* S1). To determine the baseline force–[Ca^2+^]_i_ relationship for each fibre at the start of the experiment, fibres in the two isometric groups were initially stimulated to produce contractions at 15–150 Hz (350 ms duration) at 1 min intervals, and force and [Ca^2+^]_i_ were recorded. After 5 min of rest, fatiguing stimulation commenced. Fibres in the low‐intensity group were given submaximal 50 Hz tetani of 350 ms duration every 5 s for 100 contractions.^31^ In the high‐intensity group, fibres were exposed to six sets of 30 s contraction bouts consisting of 250 ms, 100 Hz contractions given at 500 ms interval with 4.5 min rest between bouts.^25^ After 30 min recovery, fibres were stimulated at low frequency (30 Hz) and at high frequency (120 Hz) to determine the extent of PLFFD. Thereafter, fibres were treated for 5 min with CK‐2066260 (10 μM) and then stimulated again at 30 and 120 Hz to determine the extent to which CK‐2066260 counteracted PLFFD. No control experiments were performed to assess a potential increase in force between 30 and 35 min of recovery without exposure to CK‐2066260 because previous studies show that fibres are in a stable state of PLFFD during this time period, which means that no or very minor changes in force would be expected.^21^, ^35^

Flexor digitorum brevis muscle single fibres or small fibre bundles were exposed to repeated eccentric contractions after adjusting to optimal length for force production. The baseline isometric force at 30 and 120 Hz was measured during 350 ms contractions with 1 min of rest between contractions. Thereafter, the repeated lengthening contraction protocol commenced with 100 Hz tetani (350 ms duration) given once every 20 s for a total of 10 contractions. A 20% ramp length change was performed during the final 150 ms of each contraction.^27^ Finally, force was measured during 30 and 120 Hz isometric tetani after 30 min recovery in standard Tyrode and after additional 5 min in the presence of CK‐2066260 (10 μM). [Ca^2+^]_i_ was not measured in the eccentric contraction experiments because we recently showed that tetanic [Ca^2+^]_i_ was only marginally affected by a much more severe protocol with 100 repeated eccentric contractions.^27^

### Statistics

Statistical evaluation employed repeated measures analysis of variance using GraphPad Prism 8.1.2 software (GraphPad, San Diego, CA, USA). *Post hoc* analysis was performed using either Tukey's or Dunnett's test. Data are presented as mean ± standard error of the mean. Statistically significant differences were determined at *P* < 0.05.

## Results

### Low‐intensity isometric contractions

**FIGURE 1:**
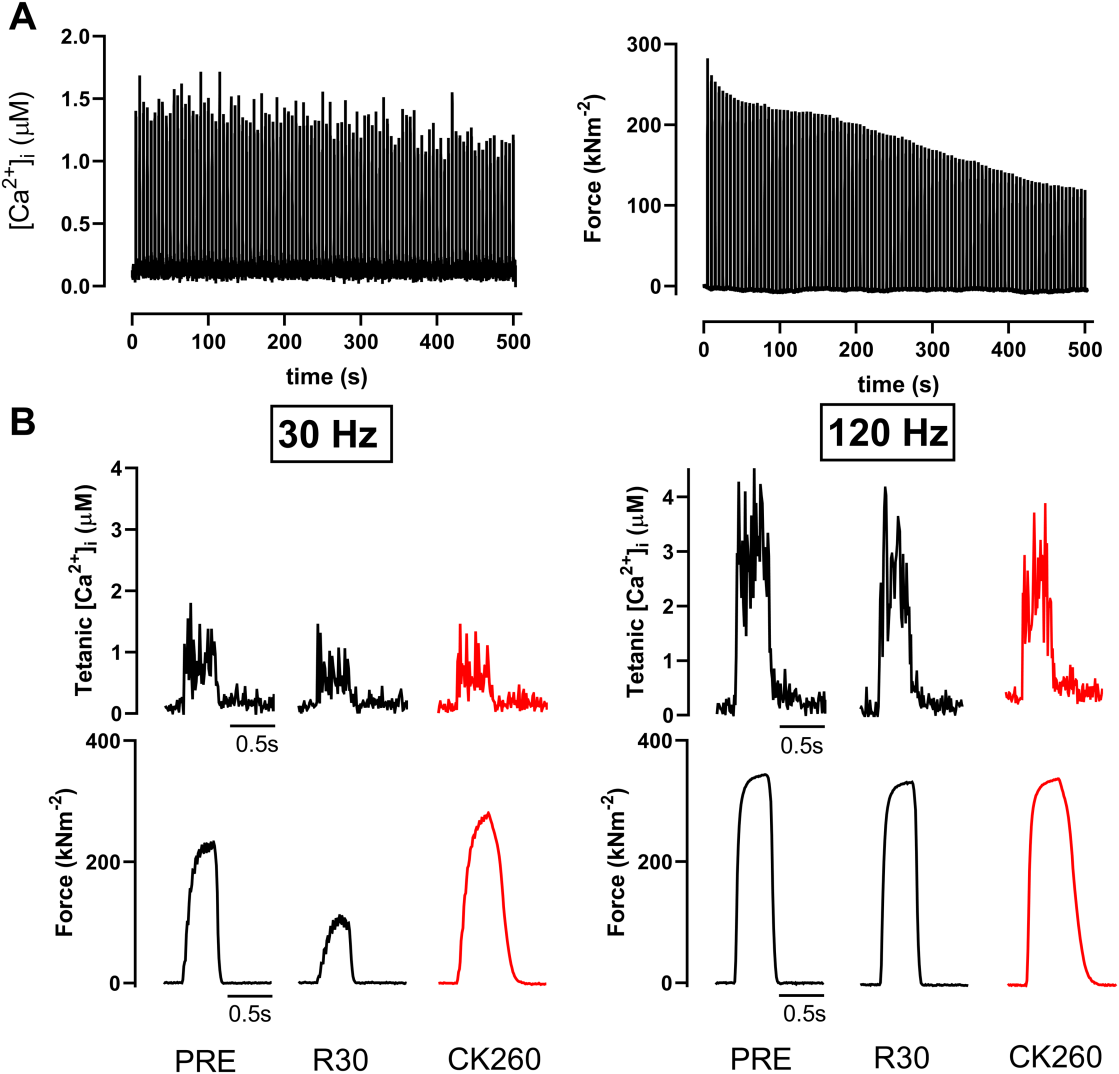
CK‐2066260 increases low‐frequency force during the prolonged low‐frequency force depression caused by low‐intensity contractions. (A) Typical recordings of [Ca^2+^]_i_ and force during repeated low‐intensity stimulation (50 Hz tetani of 350 ms duration given every 5 s for 100 contractions) in an isolated flexor digitorum brevis fibre. (B) Typical example of 30 and 120 Hz tetanic [Ca^2+^]_i_ and force elicited in the unfatigued state (PRE), at 30 min of recovery (R30), and after an additional 5 min superfusion with 10 μM of CK‐2066260 (CK260).

**FIGURE 2:**
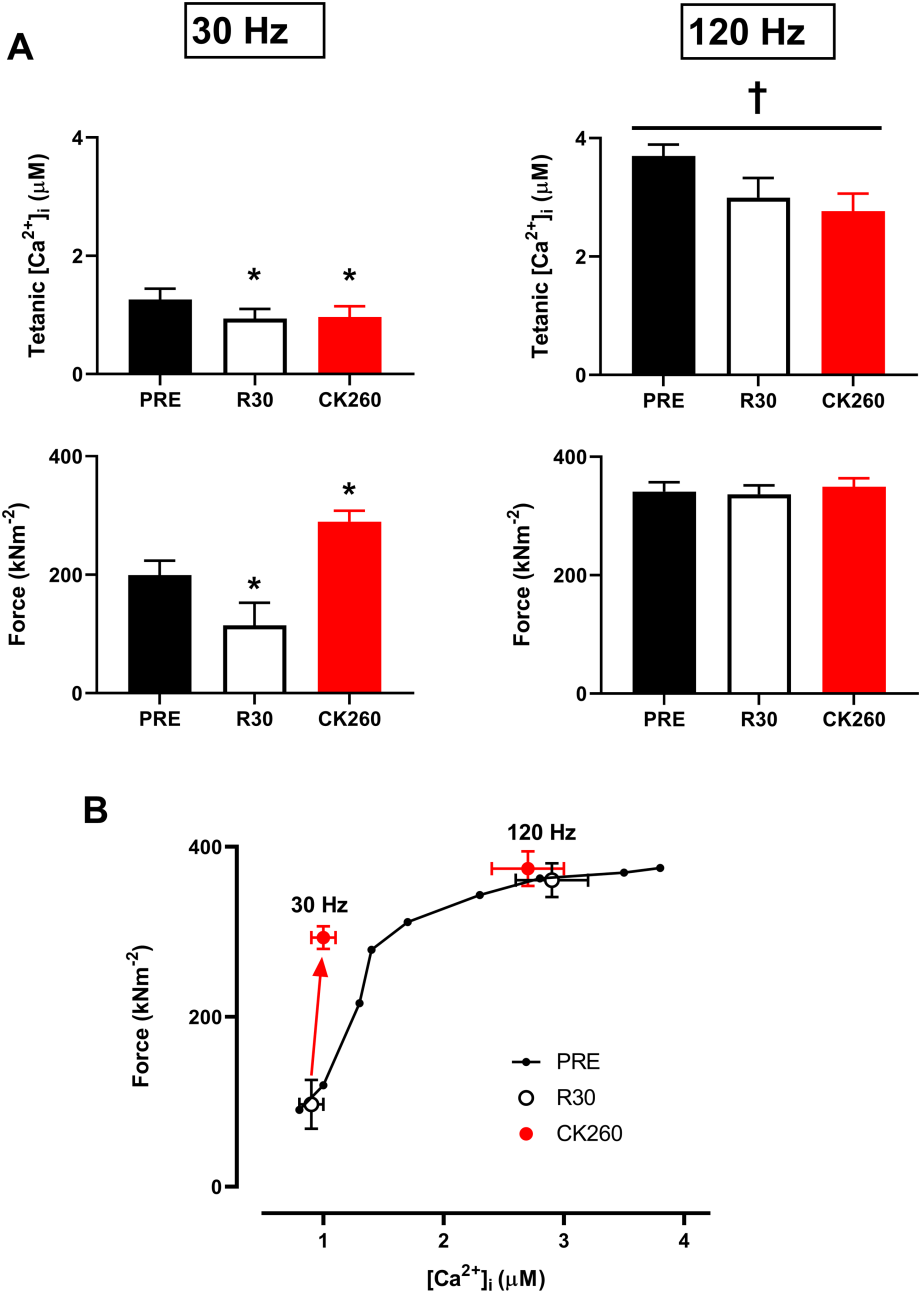
CK‐2066260 reverses prolonged low‐frequency force depression after low‐intensity contractions. (A) Mean values of 30 and 120 Hz tetanic [Ca^2+^]_i_ and force elicited in the unfatigued state (PRE), at 30 min of recovery (R30), and after an additional 5 min superfusion with 10 μM of CK‐2066260 (CK260). (B) Mean force–[Ca^2+^]_i_ relationship obtained from 15 to 150 Hz stimulations in the unfatigued state (PRE), and 30 and 120 Hz force and tetanic [Ca^2+^]_i_ elicited at 30 min of recovery (R30), and after 5 min superfusion with 10 μM CK‐2066260. Data shown as mean ± standard error of the mean (*n* = 5). ^*^Significantly different from PRE at *P* < 0.05. ^†^Significant main effect with depressed 120 Hz tetanic [Ca^2+^]_i_ during prolonged low‐frequency force depression at *P* < 0.05, whereas the *post hoc* test did not show any significant difference between the individual groups.

The low‐intensity isometric contractions protocol resulted in marked fatigue with the force in the last of the 100 tetani being reduced to 47 ± 4% of the force in the first tetanus (*n* = 7). This force decrease was associated with a progressive decline in mean tetanic [Ca^2+^]_i_ to 76 ± 13% of the initial value (*Figure* 1A). At 30 min post‐fatigue, the extent of PLFFD was assessed by stimulating at low (30 Hz) and high (120 Hz) frequencies with PLFFD typically exemplified by a more pronounced force depression at the lower stimulation frequency. Indeed, PLFFD was induced by the low‐intensity fatigue protocol with decreased 30 Hz force and tetanic [Ca^2+^]_i_, whereas there was no significant reduction in 120 Hz force (*Figures* 1B and 2A). The major cause of PLFFD following low‐intensity contractions was reduced SR Ca^2+^ release; although both 30 Hz force and [Ca^2+^]_i_ at 30 min recovery were decreased, the relation between them still lies near the unfatigued force–[Ca^2+^]_i_ relationship (*Figure* 2B). We also observed a decrease in 120 Hz tetanic [Ca^2+^]_i_ (*Figures* 1B and 2A), but it did not cause a force reduction because tetanic [Ca^2+^]_i_ was large enough to reach the plateau of the force–[Ca^2+^]_i_ relationship where changes in [Ca^2+^]_i_ have little impact on force production (*Figure* 2B). PLFFD was fully reversed following 5 min superfusion with CK‐2066260. In fact, 30 Hz force in the presence of CK‐2066260 was larger than at baseline (PRE), and this occurred without any significant alteration in [Ca^2+^]_i_ (*Figures* 1B and 2). On the other hand, 120 Hz force and [Ca^2+^]_i_ were not affected by CK‐2066260 treatment. Thus, PLFFD following low‐intensity stimulation was caused by decreased SR Ca^2+^ release and CK‐2066260 reversed PLFFD by increasing myofibrillar Ca^2+^ sensitivity.

### High‐intensity isometric contractions

**FIGURE 3:**
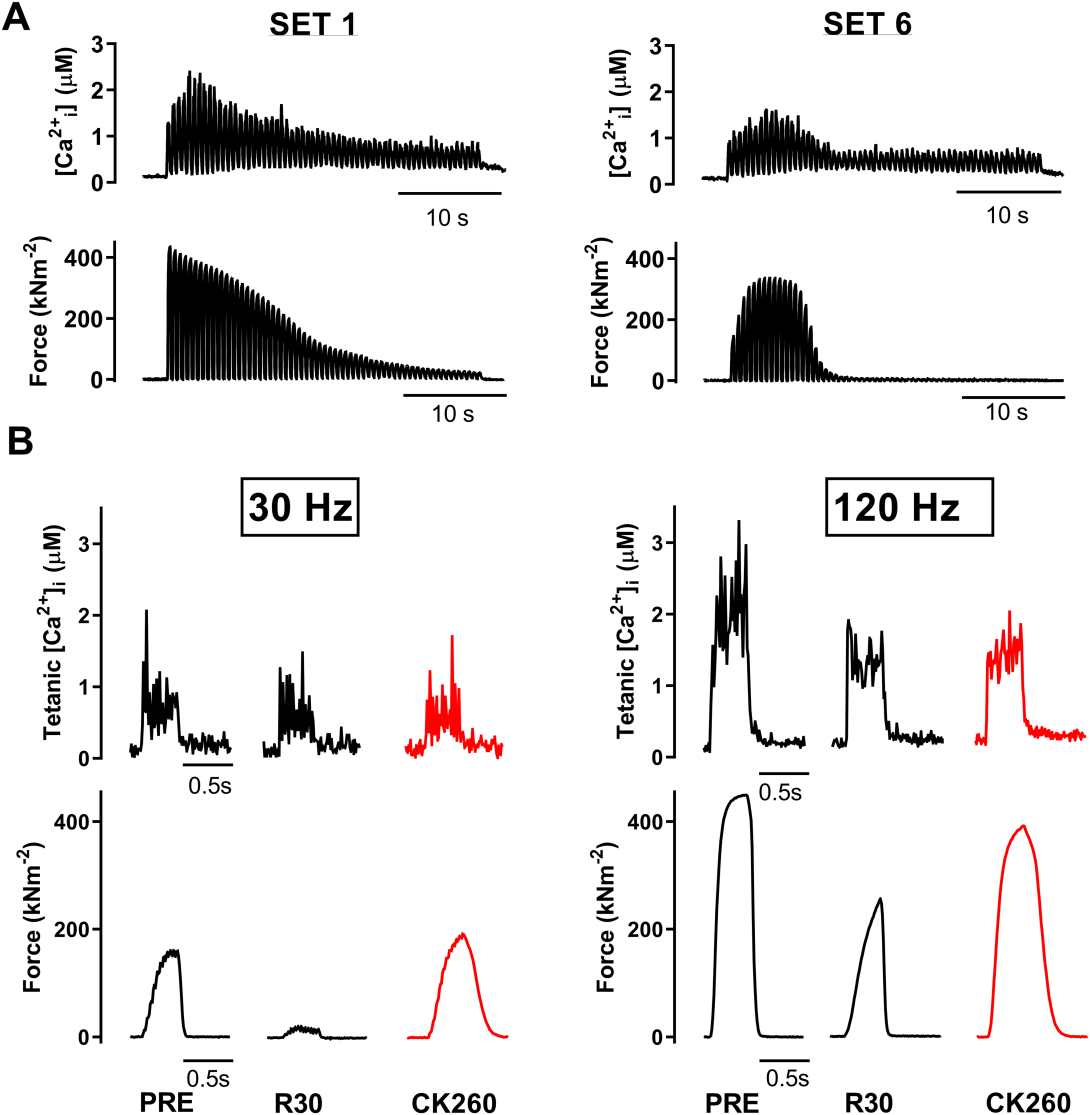
CK‐2066260 increases force during prolonged low‐frequency force depression caused by high‐intensity stimulation. (A) Typical recordings of [Ca^2+^]_i_ and force during the first and final (sixth) set of high‐intensity stimulation (100 Hz tetani of 250 ms duration given every 0.5 s for 60 contractions) in an isolated flexor digitorum brevis fibre. (B) Representative traces of 30 and 120 Hz tetanic [Ca^2+^]_i_ and force elicited in the unfatigued state (PRE), at 30 min of recovery (R30), and after an additional 5 min of superfusion with 10 μM CK‐2066260 (CK260). Intriguingly, the almost completely abolished 30 Hz force at R30 is reversed by CK‐2066260 treatment by increasing myofibrillar Ca^2+^ sensitivity with no alteration of tetanic [Ca^2+^]_i_.

**FIGURE 4:**
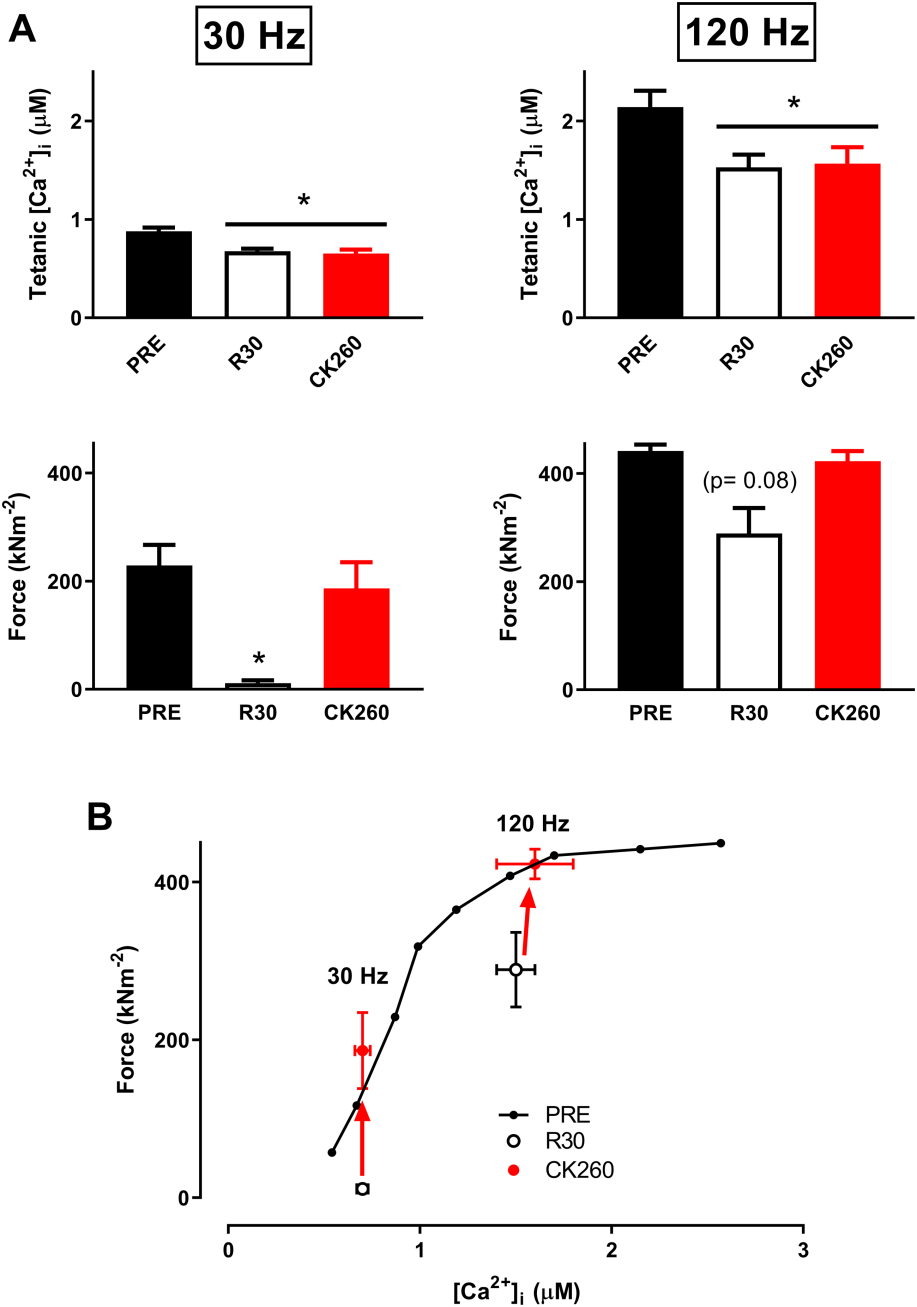
CK‐2066260 reverses the severe prolonged low‐frequency force depression induced by high‐intensity contractions. (A) Mean values of 30 and 120 Hz tetanic [Ca^2+^]_i_ and force elicited in the unfatigued state (PRE), at 30 min of recovery (R30), and after an additional 5 min superfusion with 10 μM CK‐2066260 (CK260). (B) Mean force–[Ca^2+^]_i_ relationship obtained from 15 to 150 Hz stimulations in the unfatigued state (PRE), and mean 30 and 120 Hz force–[Ca^2+^]_i_ values obtained at 30 min of recovery (R30), and after an additional 5 min superfusion with 10 μM CK‐2066260. CK‐2066260 fully recovers force by markedly increasing myofibrillar Ca^2+^ sensitivity, while tetanic [Ca^2+^]_i_ remains unaffected. Data shown as mean ± standard error of the mean (*n* = 5). ^*^Significantly different from value at PRE at *P* < 0.05.

Next, we used a stimulation paradigm that mimicked high‐intensity exercise in humans involving six sets of high‐frequency contractions given at a 50% duty cycle for 30 s.^27^ The typical [Ca^2+^]_i_ and force records in *Figure* 3A show that severe fatigue developed during each set of contractions and that partial recovery took place during the resting period between sets. A marked PLFFD was observed 30 min after cessation of these high‐intensity contractions with 30 Hz force being decreased to <10% of the unfatigued values (*Figures* 3B, 4A, and 4B). We also observed a decrease in [Ca^2+^]_i_ during contractions, but the force reduction was much larger than what could be explained by the decrease in [Ca^2+^]_i_. This means that in addition to decreased SR Ca^2+^ release, the high‐intensity stimulation also resulted in a marked decrease in myofibrillar Ca^2+^ sensitivity, which was manifested as a substantial rightward shift in the force–[Ca^2+^]_i_ relationship (*Figure* 4B). Nevertheless, 5 min exposure to CK‐2066260 reversed PLFFD, and this occurred without any significant effect on tetanic [Ca^2+^]_i_ (*Figures* 3B and 4).

### Eccentric contractions

**FIGURE 5:**
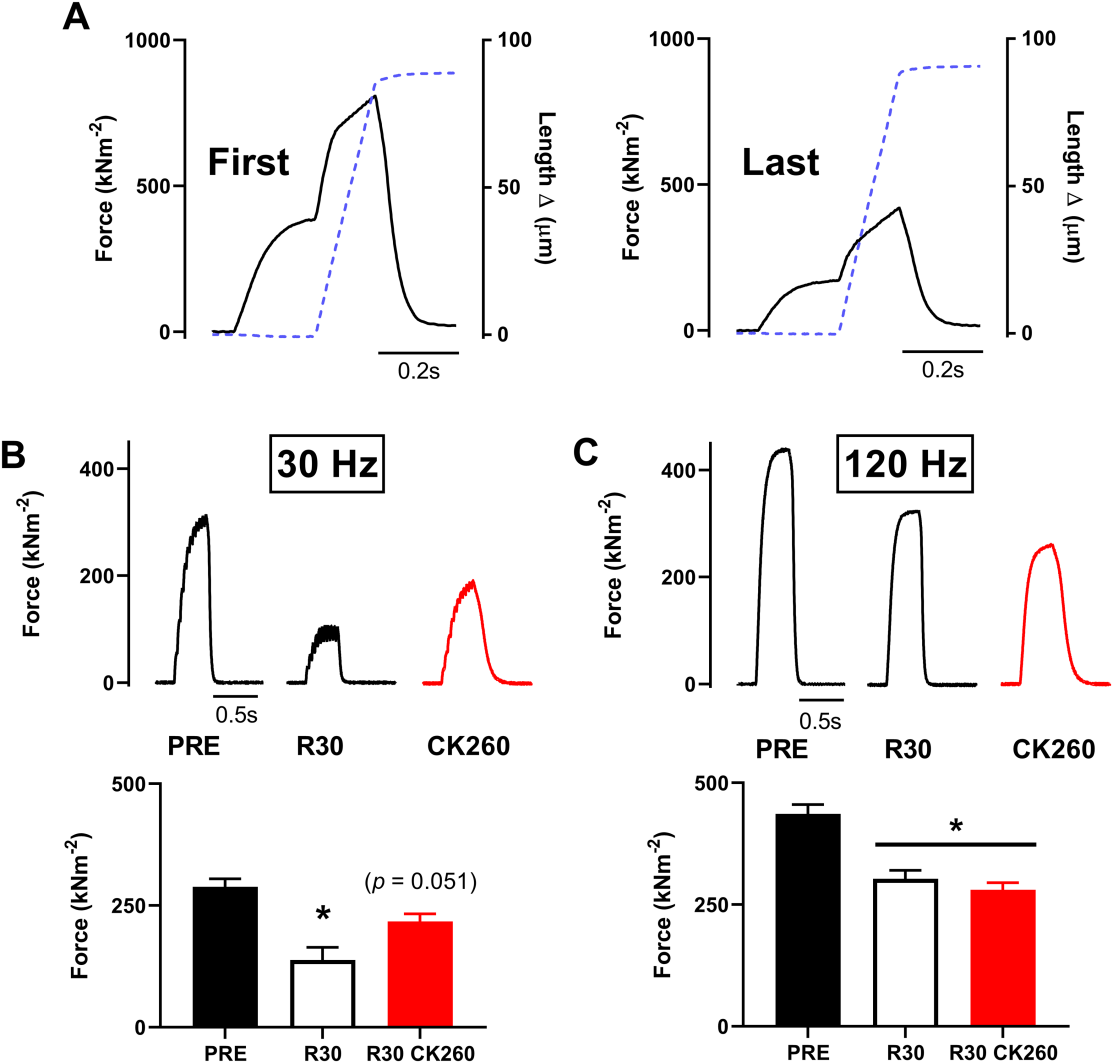
Prolonged low‐frequency force depression induced by repeated lengthening contractions is partially ameliorated by CK‐2066260 superfusion. (A) Typical force recordings from the first and last (10th) 100 Hz tetanus involving an initial isometric contraction at optimal length with a 20% lengthening step given midway during the contractions (dashed blue line). Representative traces and mean values (±standard error of the mean, *n* = 5) of (B) 30 Hz and (C) 120 Hz force elicited in the unfatigued state (PRE), at 30 min of recovery (R30), and after an additional 5 min superfusion with 10 μM CK‐2066260 (CK260). ^*^Significantly different from value at PRE at *P* < 0.05.

Prolonged low‐frequency force depression can be observed following repeated eccentric contractions with myofibrillar damage as a dominant cause of PLFFD.^26^, ^27^, ^37^ In a final set of experiments, FDB fibres or small fibre bundles were exposed to eccentric 100 Hz tetani at long (20 s) rest periods between contractions. These long rest periods were applied in order to minimize fatigue development due to metabolic alterations while allowing for induction of impaired myofibrillar function with the repeated lengthening contractions. Typical force traces from the first and last (10th) contractions show that the repeated eccentric contractions resulted in ~50% decrease in isometric force (i.e. before the start of lengthening) and peak force during the eccentric phase (*Figure* 5A). After 30 min of recovery, PLFFD was evident with a greater relative reduction in 30 Hz than in 120 Hz tetanic force (*Figure* 5B and 5C). Five minute superfusion with CK‐2066260 partially recovered 30 Hz force, whereas 120 Hz force was not improved. Thus, while CK‐2066260 proved beneficial in partially reversing PLFFD following repeated eccentric contractions, it could not compensate for the impaired cross‐bridge force‐generating capacity manifested as decreased near‐maximal 120 Hz force (*Figure* 5C).

## Discussion

Skeletal muscle weakness, premature fatigue development, and exercise intolerance are important features in numerous pathological conditions.^38^, ^39^ These muscle deficiencies can also develop as a consequence of a lifestyle with limited physical activity,^40^ as part of the ageing process,^41^ or in mechanically ventilated intensive care unit patients.^42^, ^43^ Here, we used PLFFD induced by three types of exercise as models of muscle weakness with different underlying mechanisms. The results show that the FSTA CK‐2066260 fully reversed muscle weakness following low‐intensity and high‐intensity exercise, while it was only partially effective in reversing the weakness induced by damaging eccentric contractions.

Skeletal muscle fatigue induced by low‐intensity to moderate‐intensity endurance‐type exercise has been related to intramuscular glycogen depletion and impaired SR Ca^2+^ release.^44^, ^45^, ^46^, ^47^, ^48^ Thus, the recovery of SR Ca^2+^ release and force following prolonged endurance exercise will largely depend on the rate of glycogen re‐synthesis,^35^ which is slow, with up to 24 h potentially required for glycogen to be fully restored.^49^, ^50^ The low‐intensity stimulation protocol used in the present study has been shown to cause glycogen depletion and decreased SR Ca^2+^ release.^51^ Accordingly, PLFFD after the low‐intensity stimulation can be fully ascribed to decreased [Ca^2+^]_i_ during contractions (see *Figure* 2). The FSTA CK‐2066260 reversed PLFFD by increasing myofibrillar Ca^2+^ sensitivity, which fully compensated for the decreased SR Ca^2+^ release. In fact, submaximal forces in the presence of the FSTA were even larger than in the unfatigued state. Translated to the *in vivo* situation, this means that the same muscular activity can be performed at a decreased neuronal activation (i.e. fewer motor units activated and/or reduced frequency of action potentials in active motor units), which will facilitate physical activities in people suffering from muscle weakness. Moreover, in the presence of the FSTA, less SR Ca^2+^ release is required to produce the same force, which might further improve the performance during endurance‐type activities by reducing the energy consumed by the ATP‐driven SR Ca^2+^ reuptake.^31^ A potential concern with FSTA treatment is that the rate of force relaxation will decrease, which might impair performance during activities requiring rapidly alternating movements.^29^ However, no limitations in coordination or voluntary exercise performance were apparent in CK‐2066260‐treated rats; rather, they showed better *in vivo* rotarod and treadmill running performance than vehicle‐treated rats.^31^

Although fatigue from high‐intensity exercise is also associated with metabolic stress that causes decreased SR Ca^2+^ release, decreased myofibrillar Ca^2+^ sensitivity, and decreased myofibrillar force,^52^ these metabolic changes including metabolite clearance of inorganic phosphate ions and phosphocreatine re‐synthesis are readily reversed within 5 min post‐exercise.^53^ This implicates other factors being responsible for induction of PLFFD after high‐intensity exercise. For instance, high‐intensity contractions cause large increases in the production of reactive oxygen and nitrogen species within skeletal muscle,^25^, ^54^ and these highly reactive molecules have been shown to induce long‐lasting effects on SR Ca^2+^ release and myofibrillar contractile function.^20^, ^55^, ^56^, ^57^ Still, antioxidants have largely been unsuccessful in reversing PLFFD because submaximal force depends on a fine‐tuned, and not fully characterized, redox balance where both severely reduced and oxidized states within skeletal muscle can depress contractile force.^22^, ^24^, ^58^ Here, we show that treatment with a FSTA effectively increases forces up to the unfatigued level during PLFFD induced by high‐intensity contractions (see *Figure* 4), and this is achieved without any increase in SR Ca^2+^ release or restoration of redox‐induced modifications involved in the development of this type of weakness.

During repeated eccentric contractions, the high forces produced during lengthening are known to induce mechanical damage to the myofibrillar contractile machinery, but the exact mechanisms underlying the decreased force‐generating capacity are still uncertain.^27^, ^37^, ^59^, ^60^ Although the force reduction after damaging eccentric contractions is larger at low than at high stimulation frequencies (i.e. PLFFD is present), force is also depressed at high stimulation frequencies, hence reflecting a decrease in the maximum force‐generating capacity.^19^, ^27^ Our present results show that while application of FSTA CK‐2066260 effectively increases the force at low (30 Hz) stimulation, it is ineffective at (120 Hz) stimulation (see *Figure* 5). This latter finding is expected because increasing the myofibrillar Ca^2+^ sensitivity would not increase the force produced by maximally Ca^2+^‐activated myofibrils.

In conclusion, we here demonstrate that the FSTA, CK‐2066260, effectively increases submaximal force in conditions with muscle weakness, and this force‐potentiating effect is not restricted to a specific mechanism underlying the weakness. Thus, FSTAs have the potential to markedly improve physical performance in humans where everyday activities are limited by muscle weakness, premature fatigue, and exercise intolerance. To this date, the FSTAs *tirasemtiv* and *reldesemtiv* have been shown to amplify the skeletal muscle force response to nerve stimulation in healthy human subjects,^61^, ^62^ and FSTAs merit further clinical trial investigation to better identify its potential to improve muscle function in conditions characterized by muscle weakness and fatigue.

## Conflict of interest

D.T.H and F.I.M. are current employees of Cytokinetics, Inc. and were compensated financially for their work. The laboratory of H.W. received financial support from Cytokinetics, Inc.

## Funding

The study was supported by grants from the Swedish Research Council (Vetenskapsrådet, H.W., 2018‐02576) and the Swedish Research Council for Sport Science (A.J.C., FO2018‐0019; H.W., P2019‐0060).

## Supporting information


**Figure S1.** Schematic of the three experimental protocols. CK‐2066260 (CK260), prolonged low‐frequency force depression (PLFFD).
