# Supplementary figures and images for: Fast skeletal muscle troponin activator CK‐2066260 mitigates skeletal muscle weakness independently of the underlying cause
Source: J Cachexia Sarcopenia Muscle. 2020 Sep 21;11(6):1747–57. doi: 10.1002/jcsm.12624 (PMC7749611; doi:10.1002/jcsm.12624)

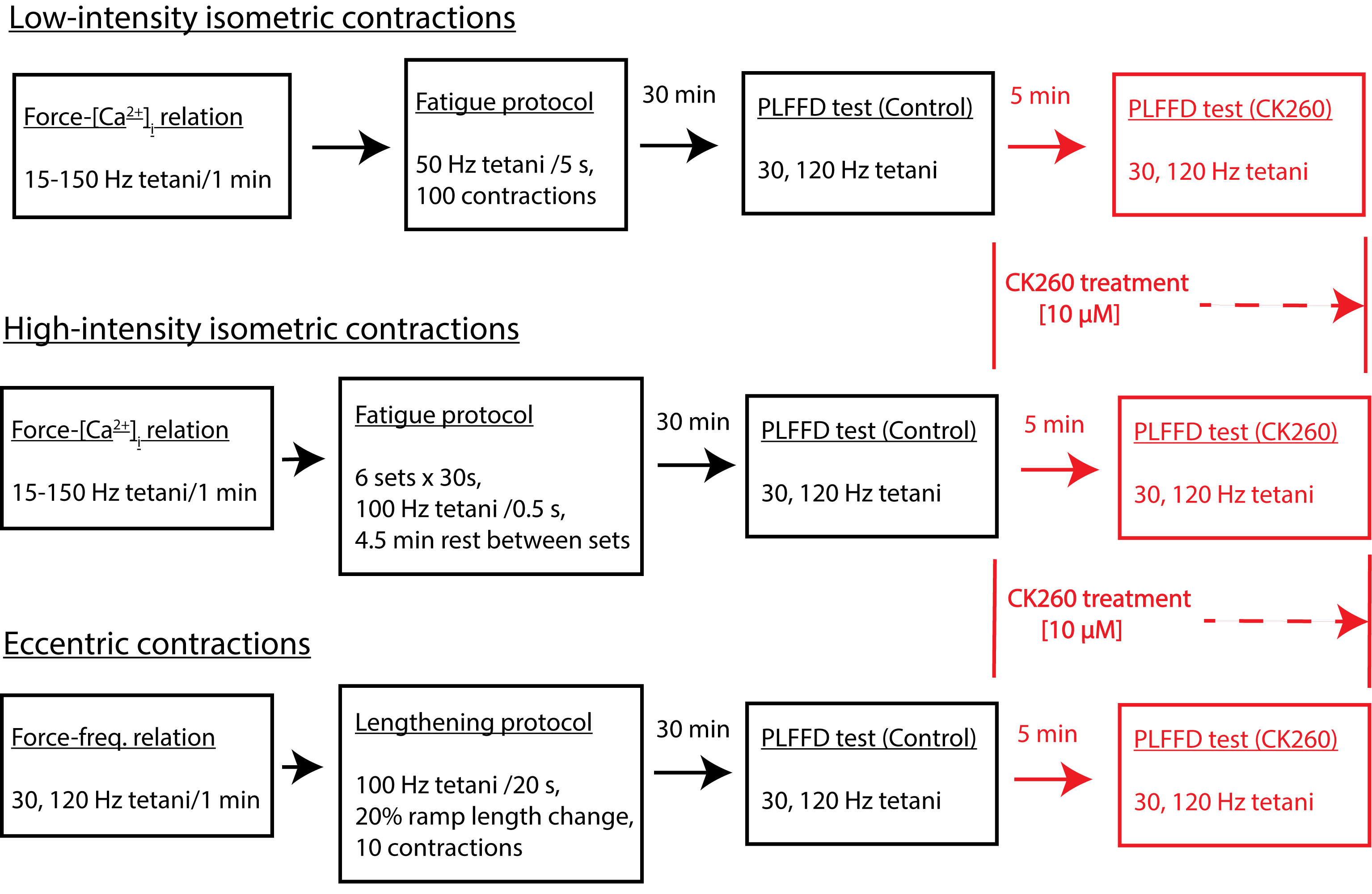

Supplement: Supplementary file 1 — Figure S1. Schematic of the three experimental protocols. CK‐2066260 (CK260), prolonged low‐frequency force depression (PLFFD). [file JCSM-11-1747-s001.tif]
